# Alexithymia, Resilience, and Depression in Functional Impairment Among Adolescents With Eating Disorders: A Case-Control Study

**DOI:** 10.31083/AP45458

**Published:** 2026-02-26

**Authors:** Yavuz Meral, Saliha Açıkgöz, Alperen Bıkmazer, Büşra Arslan, Oğuzhan Koyuncu, Melike Ayşegül Kara Karaman, Vahdet Görmez

**Affiliations:** ^1^Department of Child and Adolescent Psychiatry, School of Medicine, Istanbul Medeniyet University, 34865 Istanbul, Türkiye; ^2^Department of Child and Adolescent Psychiatry, Basaksehir Cam and Sakura City Hospital, 34480 Basaksehir, Türkiye; ^3^Department of Adult Psychiatry, Cubuk Halil Sivgin State Hospital, 06760 Ankara, Türkiye; ^4^Department of Child and Adolescent Psychiatry, Sabuncuoglu Serafeddin Training and Research Hospital, 05100 Amasya, Türkiye; ^5^Child and Adolescent Psychiatry, Goztepe Prof. Dr. Suleyman Yalcin City Hospital, 34722 Istanbul, Türkiye; ^6^College of Islamic Studies, Hamad Bin Khalifa University, 34110 Doha, Qatar

**Keywords:** alexithymia, depressive disorder, feeding and eating disorders, psychosocial functioning, psychological resilience

## Abstract

**Background:**

Eating disorders (EDs) in adolescence are associated with marked emotional difficulties and broad functional impairment. The objective of this study was to examine the relationships among alexithymia, resilience, internalizing symptoms (anxiety and depression), and functional impairment in adolescents with EDs.

**Method:**

A cross-sectional, case-control study included 51 adolescents diagnosed with EDs according to Diagnostic and Statistical Manual of Mental Disorders, 5th Edition criteria (primarily anorexia nervosa, bulimia nervosa, and binge eating disorder) and 57 matched controls. Adolescents completed self-report measures assessing alexithymia, resilience, and internalizing symptoms, and parents reported on functional impairment.

**Results:**

Adolescents with EDs showed significantly greater functional impairment at both global and subdomain levels, along with higher levels of depressive and anxiety symptoms, higher alexithymia scores, and lower resilience than did controls. Difficulty describing feelings (DDF) was the only variable significantly associated with ED group membership. Within the ED group, functional impairment was positively correlated with depressive symptoms, anxiety symptoms, DDF, and difficulty identifying feelings (DIF), and was negatively correlated with resilience. Depressive symptoms were significantly associated with functional impairment, and mediation analyses indicated that the association between DIF and global functional impairment was statistically mediated by depressive symptoms.

**Conclusion:**

These findings suggested that adolescents with EDs experience widespread functional difficulties, with depressive mood, exacerbated by challenges in emotion identification, potentially contributing to these impairments.

## Main Points

1. Eating disorders during adolescence are linked to substantial emotional difficulties and widespread functional impairment.

2. Adolescents with eating disorders showed greater functional impairment, higher alexithymia, lower resilience, and more severe depressive and anxiety symptoms than controls.

3. Difficulty describing feelings was the only factor that independently distinguished adolescents with eating disorders from controls, and depressive symptoms were the only independent factor associated with functional impairment.

4. This study provides novel evidence that depressive symptoms play a central role in the statistical link between emotion-processing difficulties and functional impairment in adolescents with eating disorders.

## 1. Introduction

Eating disorders (EDs), including anorexia nervosa (AN), bulimia nervosa (BN), and binge-eating disorder (BED) are severe psychiatric conditions that typically emerge during adolescence, a developmental stage marked by rapid biological changes, identity formation, and increased social sensitivity [1, 2]. In addition to significant disturbances in eating behavior and body image, EDs are associated with a broad spectrum of medical complications, intense psychological distress, and substantial impairments in social, academic, and occupational functioning [2, 3, 4, 5]. Global-prevalence estimates have suggested that EDs affect approximately 8.4% of women and 2.2% of men, with peak onset occurring in mid-adolescence and early adulthood [6].

Functional impairment refers to a reduction or loss of an individual’s ability to fulfill essential roles and responsibilities in daily life, including notable difficulties in areas such as social relationships, self-care, education, occupational activities, and leisure pursuits [7]. It reflects not merely the presence of symptoms, but their tangible impact on everyday functioning and social participation, and is recognized as a core diagnostic criterion for many psychiatric disorders in the Diagnostic and Statistical Manual of Mental Disorders, 5th Edition (DSM-5) [8]. Notably, studies examining functional impairment in adolescents with EDs, especially those involving comparisons with control groups, remain limited. A longitudinal, community-based epidemiological study in adolescent girls found that individuals later diagnosed with an ED typically experienced greater functional impairment, increased suicidality, and higher utilization of mental health services [2]. Similarly, research involving young adult samples has identified impaired interpersonal functioning as a transdiagnostic risk factor for EDs [5], and has further demonstrated that EDs are associated with reduced quality of life [9, 10, 11]. Although many studies have explored the adverse associations between ED symptoms and functional impairment, domain-specific comparisons with control groups and their relevance to alexithymia, particularly in adolescent populations, are still scarce [12, 13, 14, 15, 16, 17].

Alexithymia refers to difficulties in identifying and expressing one’s emotions and is characterized by a “stimulus-bound, externally oriented cognitive style” with limited imagination and introspection [18]. It is one of the most frequently studied transdiagnostic socioemotional constructs in the ED literature and represents a core structural component of ED psychopathology [19, 20]. Alexithymia’s transdiagnostic nature positions it as a shared feature across various psychiatric conditions, and also emphasizes its relevance within specific ED subtypes. A meta-analysis has shown that alexithymia is consistently elevated across ED subgroups—including AN, BN, and BED—with particularly pronounced difficulties in identifying and describing feelings [19, 21]. Strong evidence from multiple studies has indicated that alexithymia is closely linked to both the core symptoms and severity of EDs, thereby underscoring its central role in ED phenomenology [22, 23, 24, 25]. Alexithymia has been associated not only with more severe disordered eating behaviors in clinical samples, but also with elevated levels in non-clinical adolescent populations [26, 27]. Given that ED symptom severity is also related to functional impairment, there is a clear need for studies that directly examine the relationship between alexithymia and functional outcomes in this population [4]. Clarifying how distinct dimensions of alexithymia uniquely contribute to functional impairment in EDs, despite their transdiagnostic nature, may enhance our understanding of disorder-specific mechanisms. Clinically, such insights could inform targeted interventions by identifying the emotional processing issues that are most critical to improving everyday functioning.

Alexithymia is associated not only with the severity of EDs but also with depression, as demonstrated by a meta-analysis that reported moderate correlations between difficulties in identifying and describing feelings and depressive symptoms [28]. Alexithymia may function as a precursor to depression or, conversely, may arise as a consequence of diminished emotional awareness. The high comorbidity with anxiety and depression further complicates the clinical presentation of EDs [21, 28, 29, 30]. Functional impairment resulting from ED symptoms has been shown to predict later depressive symptoms, underscoring the multidimensional and intricately interrelated nature of depression, anxiety, and ED symptoms [15, 31]. However, there is a notable lack of studies, particularly those involving adolescent samples, that examine the relationship between alexithymia and functional impairment in EDs. Given the substantial role of depression in contributing to functional impairment across psychiatric conditions, it is crucial to clarify the pathways through which alexithymia influences functional outcomes in adolescents with EDs. From a theoretical perspective, disentangling the respective roles of alexithymia and depression in shaping impairment may help identify distinct mechanisms, and elucidating these differences remains a key objective.

Resilience—the capacity to adapt successfully under stress—is recognized as a potential protective factor against the risk of EDs, as supported by findings across broader psychopathology [32, 33, 34]. Psychological resilience has been associated with more favorable outcomes in EDs; higher baseline resilience predicted lower risk and improved recovery [35, 36, 37, 38, 39, 40, 41]. Profiles characterized by low resilience regulation have been linked to increased rates of EDs, depression, and anxiety [38]. Collectively, these findings point to the critical role of resilience as a transdiagnostic protective factor that not only distinguishes clinical from non-clinical populations but also influences the trajectory and recovery potential of EDs. However, research on resilience in the context of EDs has been predominantly limited to adult samples and rarely includes comparisons with control groups. Notably, to our knowledge, no study has specifically investigated whether resilience plays a protective role in functional outcomes among individuals with EDs.

Although alexithymia is widely recognized as a transdiagnostic construct, evidence has suggested that it plays a particularly central role in EDs, with important clinical implications. Nevertheless, no previous research has directly examined its association with functional impairment in adolescents with EDs, which is a critical gap, given that functional outcomes represent some of the most tangible consequences of psychiatric disorders. Previous studies consistently reported elevated levels of difficulty identifying feelings (DIF) and difficulty describing feelings (DDF) in individuals with EDs; therefore, investigating whether these dimensions are also linked to functional impairment may offer preliminary insights into disorder-specific pathways and inform refinements to etiological models. Clinically, such insights could guide interventions by identifying which facets of emotional processing are most critical for improving everyday functioning. Because of the strong association between alexithymia and depression, and the established role of internalizing symptoms in functional impairment, it is essential to determine whether observed relationships reflect direct effects or are better explained by depressive symptoms. Taken together, integrating alexithymia, internalizing symptoms, and resilience within a unified analytic framework presents a novel opportunity to advance both transdiagnostic and disorder-specific understanding, and to inform clinical strategies aimed at improving functional outcomes in adolescents with EDs. The present study was therefore designed with two primary aims: (1) to compare adolescents with EDs and a control group in functional impairment, alexithymia, resilience, anxiety, and depression; and (2) to investigate factors associated with functional impairment within the ED group, including the possible mediating role of depressive symptoms.

## 2. Materials and Methods

### 2.1 Study Design and Settings

This study was conducted as a cross-sectional, case-control, single-center investigation at the Basaksehir Cam and Sakura City Hospital, Department of Child and Adolescent Psychiatry, between June 1, 2022, and June 1, 2023.

### 2.2 Participants

The study sample comprised 108 adolescents aged 12 to 17 years, divided into two groups: a clinical group of patients diagnosed with EDs (*n* = 51) and a control group of adolescents with no reported current or past psychiatric diagnosis (*n* = 57). The mean age was 15.16 years (*SD* = 1.50) in the clinical group and 14.67 years (*SD* = 1.85) in the control group (*p* = 0.137). Most participants in both groups were female, accounting for 94.1% (48/51) in the clinical group and 84.2% (48/57) in the control group (*p* = 0.165).

The clinical sample consisted of adolescents diagnosed with an ED by a senior child and adolescent psychiatry specialist during routine outpatient visits, emergency department admissions, or inpatient consultations within the hospital setting. Inclusion criteria for this group were: (1) age between 12 and 17 years; (2) a clinical diagnosis of an ED, namely AN, including atypical AN, BN, or BED, established through clinical interviews conducted by a senior child and adolescent psychiatrist in accordance with DSM-5 criteria; and (3) provision of written informed consent signed by both the adolescent and their legal guardian. Exclusion criteria included a current or past diagnosis (or suspected diagnosis) of autism spectrum disorder, intellectual disability, global developmental delay, bipolar disorder, psychotic disorder, or substance use disorder, as well as any sensory (visual or auditory), motor, or neurological impairments that could interfere with the administration of assessments.

The control group consisted of adolescents aged 12 to 17 years who were siblings of patients (excluding those in the ED group) attending the same child- and adolescent-psychiatry outpatient clinics. These siblings had no current psychiatric complaints and no history of psychiatric diagnoses, as confirmed by parental report and medical records. Therefore, this control group should be considered clinically adjacent rather than a strictly “healthy control” sample; no standardized psychiatric interview was conducted. Adolescents were included if both they and their parents provided informed consent and met the eligibility criteria, which mirrored those of the clinical group.

### 2.3 Procedure

The study was approved by the Clinical Research Ethics Committee, Basaksehir Cam and Sakura City Hospital (Decision No: KAEK/2022.05.146), and all procedures were conducted in accordance with the ethical principles outlined in the Declaration of Helsinki. Written informed consent was obtained from the legal guardians of all participants, and participants provided written assent to confirm their voluntary participation.

Adolescents in the clinical group with a confirmed DSM-5 diagnosis of an ED were subsequently invited to participate in the study. Upon providing informed consent, participants completed a comprehensive battery of self-report measures assessing emotional functioning and interpersonal resources. These included the Toronto Alexithymia Scale-20 (TAS-20), the Child and Youth Resilience Measure-12 (CYRM-12), and the Revised Child Anxiety and Depression Scale-Child Version (RCADS-CV). Parents completed the Weiss Functional Impairment Rating Scale-Parent Form (WFIRS-P) and provided information regarding monthly household income. Height and weight were measured by clinicians using hospital-calibrated equipment.

Adolescents in the control group who met the inclusion criteria and their parents completed the same set of self-report instruments. No psychiatric interview was conducted with control participants; inclusion was based on parent-reported psychiatric history and absence of current complaints. Only adolescents who met DSM-5 diagnostic criteria for an ED at the time of assessment were included; those in full or partial remission or presenting with subthreshold symptoms were excluded. Groups were frequency-matched by sex. All assessments were conducted in a quiet, private room within the hospital, supervised by trained members of the research team.

### 2.4 Measurements

#### 2.4.1 Toronto Alexithymia Scale-20 (TAS-20)

The TAS-20 is one of the most widely used self-report instruments for assessing alexithymia, defined by difficulties in identifying and describing feelings and an externally oriented thinking style [42]. It consists of 20 items rated on a 5-point Likert scale ranging from 1 (“strongly disagree”) to 5 (“strongly agree”). Total scores range from 20 to 100, with higher scores indicating greater levels of alexithymia. Standard cut-off points are used for classification: ≤51 (non-alexithymic), 52–60 (borderline alexithymia), and ≥61 (alexithymic). The Turkish version has demonstrated satisfactory internal consistency, strong test–retest reliability, and good convergent validity, supporting its use in Turkish-speaking populations [43].

#### 2.4.2 Child and Youth Resilience Measure-12 (CYRM-12)

The CYRM-12 is a brief, standardized self-report instrument developed to assess resilience in children and adolescents [44]. It comprises 12 items rated on a 5-point Likert scale ranging from 1 (“not at all”) to 5 (“a lot”), with higher scores reflecting greater resilience. The total score is calculated by summing all item responses, yielding a range from 12 to 60. The Turkish adaptation of the CYRM-12 confirmed a unidimensional factor structure and demonstrated that the scale is a reliable and valid tool for assessing resilience in Turkish-speaking youth [45].

#### 2.4.3 The Revised Child Anxiety and Depression Scale-Child Version (RCADS-CV)

The RCADS-CV was originally developed by Chorpita *et al*. [46] based on DSM-IV symptoms. The RCADS-CV consists of 47 items rated on a 4-point Likert scale (0 = “never” to 3 = “always”) and assesses five domains of anxiety (separation anxiety disorder, social phobia, generalized anxiety disorder, panic disorder, obsessive-compulsive disorder) and major depressive disorder. The total anxiety score of the RCADS-CV is obtained by summing the anxiety subdomains. Raw scores are converted into T-scores based on age-group norms, providing a measure that accounts for developmental characteristics. The Turkish validity and reliability study demonstrated excellent internal consistency for the total scale and good reliability across the subscales, thereby supporting its suitability for both clinical and research applications [47].

#### 2.4.4 The Weiss Functional Impairment Rating Scale-Parent Form (WFIRS-P) 

The WFIRS-P is a parent-reported, 50-item instrument designed to assess functional impairment across six domains: family, school, life skills, self-concept, social activities, and risky behaviors. The scale yields a total functional impairment score, calculated as the mean of all subscale scores rather than raw sums. Each item is rated on a 4-point Likert scale ranging from 0 (“never or not at all”) to 3 (“very often or very much”), with an option for “not applicable”. Higher scores indicate greater levels of functional impairment. Although originally developed to assess impairment related to attention-deficit/hyperactivity disorder (ADHD), subsequent research has shown that the WFIRS-P is not specific to ADHD, as internalizing symptoms and other emotional or behavioral problems are also significantly associated with total and domain scores [48, 49]. Weiss *et al*. [7] suggested that the scale’s robustness across cultures, populations, and diagnostic categories stems from its capacity to measure core aspects of functional impairment. This positions the WFIRS-P as a transdiagnostic tool useful for understanding and differentiating impairment across various psychiatric conditions [7]. The Turkish adaptation confirmed the scale’s reliability and validity for both clinical and research applications [50]. In this study, scoring followed standardized procedures: items marked as “not applicable” were coded as missing and excluded from calculations. Both domain and total scores were computed as the mean of available items. Clinical thresholds were not applied; all scores were analyzed as continuous variables.

Comprehensive psychometric properties, including factor structures, internal consistency coefficients, and convergent validity indices, are presented in the **Supplementary Materials S1** for the instruments used in this study.

### 2.5 Data Analysis

All statistical analyses were conducted using Jamovi software (V 2.6.19; The Jamovi Project, Sydney, Australia). Prior to main analyses, assumptions of normality and homogeneity were verified, and appropriate corrections were applied for multiple comparisons (Benjamini–Hochberg false discovery rate [FDR] for *t*-tests and Holm adjustment for the analyses of covariance [ANCOVA]). Effect sizes were reported (Cohen’s *d* and partial η^2^*ₚ*), and the robustness of results was evaluated through sensitivity analyses excluding participants with clinically significant internalizing symptoms (RCADS-CV T ≥70). Descriptive statistics, including means, standard deviations, frequencies, and interquartile ranges, were computed for all study variables. Group differences were examined using independent samples *t*-tests and chi-square tests, and ANCOVA was used to control for potential confounds, with functional impairment (WFIRS-P Total) as the dependent variable. Binomial logistic regression identified predictors of group membership (ED vs. control). Within the ED group, Pearson correlations, backward multiple linear regressions, and generalized linear mediation models (GLM) with bias-corrected bootstrapping (5000 resamples) were performed. Post-hoc power analyses (G*Power 3.1, statistical power analysis software, version 3.1.9.6; Heinrich Heine University Düsseldorf, Düsseldorf, Westphalia, Germany) confirmed adequate statistical power (1–β > 0.80). All model assumptions were met unless otherwise noted. Statistical significance was set at *p* ≤ 0.05.

## 3. Results

### 3.1 Between-Group Analyses 

#### 3.1.1 Comparison of ED and Control Groups on Sociodemographic and Anthropometric Variables

Although no significant differences were found in age (*p* = 0.137) or height (*p* = 0.131), participants in the ED group had significantly lower weight (*M* = 50.43 kg, *SD* = 15.02) than did the control group (*M* = 57.65 kg, *SD* = 13.78), *t*(103) = –2.57, *p* = 0.012. Similarly, body mass index (BMI) was significantly lower in the ED group (*M* = 19.30, *SD* = 5.47) than in the control group (*M* = 21.47, *SD* = 4.64), *t*(103) = –2.20, *p* = 0.030 (see Table 1). Chi-square tests revealed no significant group differences in gender distribution, χ^2^(1) = 2.67, *p* = 0.102, or in monthly household income, χ^2^(4) = 6.01, *p* = 0.199. Regarding monthly income in the ED group, the distribution was as follows: below the minimum wage (3.9%), 1–2 times the minimum wage (19.6%), 2–3 times the minimum wage (52.9%), and more than three times the minimum wage (17.6%). In the control group, 1.8% did not report income, while the remainder were distributed as follows: below the minimum wage (1.8%), 1–2 times the minimum wage (22.8%), 2–3 times the minimum wage (35.1%), and more than three times the minimum wage (33.3%).

**Table 1.** **Comparison of groups on sociodemographic and anthropometric variables**.

Variable	Group	*n*	Mean	*SD*	t(df)	*p*
Age (years)	ED	51	15.16	1.50	t(106) = 1.50	0.137
	Control	57	14.67	1.85		
Height (m)	ED	51	1.61	0.06	t(104) = –1.52	0.131
	Control	55	1.63	0.07		
Weight (kg)	ED	51	50.43	15.02	t(103) = –2.57	0.012*
	Control	54	57.65	13.78		
BMI (kg/m^2^)	ED	51	19.30	5.47	t(103) = –2.20	0.030*
	Control	54	21.47	4.64		

*Note.* Sample sizes vary across variables due to missing data. *p < 0.05. ED, eating disorder; BMI, body mass index.

#### 3.1.2 Independent Samples *T*-Test Results for ED and Control Groups

Independent samples *t*-tests showed that the ED group reported significantly higher overall functional impairment, *t*(106) = 6.25, *p* < 0.001, *q* < 0.001, *d *= 1.21, with the largest difference in the self-perception domain, *t*(106) = 8.67, *p* < 0.001, *q* < 0.001, *d* = 1.67. Significant group differences were also found across all other WFIRS-P domains (all *q*s < 0.01). Similarly, the ED group showed higher alexithymia scores across TAS subscales (DIF, DDF, and externally oriented thinking [EOT]) and TAS total, with medium to large effects (all *q*s ≤ 0.032). Resilience scores were lower in the ED group, *t*(106) = –4.99, *p* < 0.001, *q* < 0.001, *d* = –0.96. Internalizing symptoms were elevated in the ED group, with significant differences in depression, *t*(105) = 5.29, *p <* 0.001, *q* < 0.001, *d* = 1.02, and total anxiety, *t*(104) = 3.79, *p* < 0.001, *q* < 0.001, *d* = 0.74. Complete statistical results, including mean differences, standard errors, and effect sizes, are presented in Table 2.

**Table 2.** **Group comparisons: independent samples *t*-tests**.

Variable	*t*(df)	ED mean	Control mean	Mean difference	SE difference	*p*	*q* (FDR)	95% CI	Cohen’s *d*
WFIRS-P total	6.25 (106)	0.65	0.29	0.36	0.06	<0.001***	<0.001	[0.25, 0.48]	1.21
WFIRS-P–risky behaviors	4.41 (106)	0.17	0.04	0.13	0.03	<0.001***	<0.001	[0.07, 0.19]	0.85
WFIRS-P–social activities	3.95 (106)	0.61	0.22	0.39	0.10	<0.001***	<0.001	[0.20, 0.59]	0.76
WFIRS-P–self-perception	8.67 (106)	1.88	0.56	1.32	0.15	<0.001***	<0.001	[1.02, 1.62]	1.67
WFIRS-P–life skills	5.07 (106)	0.98	0.52	0.47	0.09	<0.001***	<0.001	[0.28, 0.65]	0.98
WFIRS-P–school functioning	2.73 (106)	0.43	0.21	0.22	0.08	0.007**	0.010	[0.06, 0.38]	0.53
WFIRS-P–family functioning	4.04 (106)	0.69	0.35	0.34	0.08	<0.001***	<0.001	[0.17, 0.51]	0.78
TAS–DIF	6.82 (106)	22.14	13.09	9.05	1.33	<0.001***	<0.001	[6.42, 11.68]	1.31
TAS–DDF	6.91 (106)	16.35	10.30	6.06	0.88	<0.001***	<0.001	[4.32, 7.79]	1.33
TAS–EOT	2.24 (106)	23.39	21.77	1.62	0.72	0.027*	0.032	[0.19, 3.06]	0.43
TAS total	7.24 (106)	61.88	45.16	16.72	2.31	<0.001***	<0.001	[12.14, 21.31]	1.40
CYRM-12	−4.99 (106)	39.29	48.23	−8.93	1.79	<0.001***	<0.001	[−12.49, −5.38]	−0.96
RCADS-CV depression	5.29 (105)	69.96	54.41	15.55	2.94	<0.001***	<0.001	[9.72, 21.38]	1.02
RCADS-CV total anxiety	3.79 (104)	69.39	59.40	9.99	2.64	<0.001***	<0.001	[4.76, 15.23]	0.74

Note. *p < 0.05, **p < 0.01, ***p < 0.001; *p* values are two-tailed. *q* values represent false discovery rate (FDR) adjusted *p* values (Benjamini–Hochberg). WFIRS-P, weiss functional impairment rating scale-parent form; TAS, toronto alexithymia scale; DIF, difficulty identifying feelings; DDF, difficulty describing feelings; EOT, externally oriented thinking; CYRM-12, child and youth resilience measure-12; RCADS-CV, revised child anxiety and depression scale-child version.

#### 3.1.3 ANCOVA for WFIRS-P Total Scores

An ANCOVA was conducted to examine differences in functional impairment (WFIRS-P Total scores) between adolescents with ED and controls, which was adjusted for demographic covariates (sex, age, BMI). The overall model was significant, F_4,99_ = 10.00, *p* < 0.001. After controlling for demographics, adolescents with ED continued to show markedly greater impairment than did controls, F_1,99_ = 34.44, *p* < 0.001, η^2^_p_ = 0.258. None of the demographic covariates (sex, age, BMI) was a significant predictor (all *p*s > 0.20) (see Table 3).

**Table 3.** **ANCOVA summary for WFIRS-P total scores**.

Source	SS	df	MS	F	*p*	*η* ^2^	*η^2^_𝑝_*
Overall	3.490	4	0.872	10.00	<0.001***	—	—
Group	3.172	1	3.172	34.44	<0.001***	0.252	0.258
Age	0.112	1	0.112	1.21	0.274	0.009	0.012
BMI	0.079	1	0.079	0.85	0.358	0.006	0.009
Sex	0.128	1	0.128	1.39	0.241	0.010	0.014
Residuals	9.118	99	0.092	—	—	—	—

Note. ***p < 0.001; SS, sum of squares; MS, mean square; η^2^, eta squared; η^2^_𝑝_, partial eta squared. *p*-values adjusted using Holm correction.

#### 3.1.4 Binomial Logistic Regression Predicting Group Membership 

A binomial logistic regression was performed to assess predictors of ED group membership. Prior to running the binomial logistic regression, we tested the assumption of logit-linearity using the Box–Tidwell procedure for all continuous predictors (DIF, DDF, CYRM-12, RCADS-CV Depression, RCADS-CV Anxiety). No significant violations were detected (*p*s > 0.10). In addition, multicollinearity was assessed via Variance Inflation Factors (VIF) and tolerance statistics, all within acceptable ranges (VIFs <3.6). The model was statistically significant, χ^2^(5) = 44.3, *p* < 0.001, explaining 45.6% of the variance (Nagelkerke *R*^2^) and showing good discrimination (Area under curve [AUC] = 0.85). Among predictors, DDF was significantly associated with increased odds of being in the ED group (B = 0.16, SE = 0.08, *p* = 0.036, OR = 1.18, 95% CI [1.01, 1.37]). DIF (*p* = 0.073), resilience (CYRM-12), depression, and anxiety were not significant predictors (see Table 4).

**Table 4.** **Binomial logistic regression predicting ED group membership (*n* = 106)**.

Predictor	B	SE	Wald Z	*p*	OR	95% CI for OR	VIF	Tolerance
Intercept	−2.34	2.66	−0.88	0.379	0.10	5.27 × 10^−⁢4^–17.71	–	–
TAS–DIF	0.10	0.05	1.79	0.073	1.10	0.99–1.22	2.32	0.431
TAS–DDF	0.16	0.08	2.10	0.036*	1.18	1.01–1.37	2.00	0.500
CYRM-12	−0.02	0.04	−0.47	0.642	0.98	0.92–1.05	1.86	0.537
RCADS–CV depression	0.01	0.03	0.29	0.775	1.01	0.95–1.07	3.58	0.279
RCADS–CV total anxiety	−0.02	0.03	−0.73	0.465	0.98	0.93–1.04	2.69	0.371

Note. *p ≤ 0.05; OR, odds ratio; CI, confidence interval; VIF, variance inflation factors; AUC, area under curve; SE, standard error; Outcome coding: 1 = ED group, 0 = control group. Model fit: χ^2^(5) = 44.3, *p* < 0.001; Nagelkerke *R*^2^ = 0.456; AUC = 0.85.

#### 3.1.5 Sensitivity Analysis

3.1.5.1 Group Comparison

Group differences in functional impairment remained robust across all WFIRS-P domains, with larger effects observed for Total impairment (*d* = 1.47 vs. 1.21), Family (*d* = 0.98 vs. 0.78), Life Skills (*d* = 1.12 vs. 0.98), Self-Perception (*d* = 1.97 vs. 1.67), and Risky Behaviors (*d* = 0.97 vs. 0.85). Differences in School (*d* = 0.67 vs. 0.53) and Social Activities (*d* = 0.93 vs. 0.76) were comparable but modestly increased. For alexithymia, effect sizes were amplified, particularly for DIF (*d* = 1.68 vs. 1.31), DDF (*d* = 1.51 vs. 1.33), and TAS Total (*d* = 1.76 vs. 1.40), whereas EOT remained small (*d* = 0.52 vs. 0.43). Internalizing symptoms also showed stronger effects as expected, with RCADS-CV Depression (*d* = 1.67 vs. 1.02), and RCADS-CV Anxiety (*d* = 1.56 vs. 0.74). Resilience was consistently lower in the ED group, with a stronger effect size (*d* = –1.27 vs. –0.96). ANCOVA confirmed these patterns, with the Group effect explaining a larger proportion of variance in impairment (η^2^_𝑝_ = 0.362 vs. 0.258). See **Supplementary Material S2** for **Supplementary Tables 1,2**.

3.1.5.2 Power Analysis

A sensitivity power analysis was conducted using G*Power 3.1 to determine the minimum detectable effect size with the available sample. For independent-samples *t*-tests (*n* = 108, α = 0.05, power = 0.80), the study was adequately powered to detect medium-to-large effects (Cohen’s *d* ≥0.55). For ANCOVA models (*n* ≈ 100, 1 group factor, 3–5 covariates), the study was sufficiently powered to detect medium effects (partial η^2^ ≥ 0.06). Observed group effects on WFIRS-P Total scores (η^2^*ₚ* = 0.18–0.36) were well above this threshold, supporting the adequacy of the sample size to test the primary hypotheses.

### 3.2 Within-Group Analyses in the ED Sample

#### 3.2.1 Diagnostic Frequencies Within the Eating Disorder Sample 

Within the ED group (*n* = 51), the most prevalent diagnosis was AN, encompassing both typical and atypical forms. Specifically, 52.9% of participants (*n* = 27) were diagnosed with AN, and an additional 11.8% (*n* = 6) met criteria for atypical AN, yielding a combined prevalence of 64.7% for anorexic presentations. BN was diagnosed in 27.5% (*n* = 14) of the sample, and BED in 7.8% (*n* = 4). Of the ED group, 17.6% (*n* = 9), all diagnosed with AN, were hospitalized in the pediatric ward for medical stabilization and re-nutrition prior to psychiatric consultation.

#### 3.2.2 Correlation Analysis

Pearson correlation analyses were conducted to examine the relationships among functional impairment, alexithymia dimensions, resilience, depressive symptoms, anxiety, age, and BMI within the ED group (*n* = 51).

Functional impairment (WFIRS-P Total) was positively correlated with difficulty identifying feelings (DIF; *r* = 0.326, *p* = 0.02), difficulty describing feelings (DDF; *r* = 0.413, *p* = 0.003), and total alexithymia (TAS Total; *r* = 0.410, *p* = 0.003). No significant association was observed with externally oriented thinking (EOT; *r* = 0.135, *p* = 0.345). Functional impairment was also negatively correlated with resilience (CYRM-12; *r* = –0.308, *p* = 0.028). In addition, higher depression scores (*r* = 0.463, *p* < 0.001) and higher anxiety scores (*r* = 0.391, *p* = 0.005) were significantly associated with greater functional impairment. Neither age (*r *= 0.105, *p* = 0.464) nor BMI (*r* = 0.222, *p* = 0.117) was significantly related to functional impairment.

Depression scores were strongly correlated with DIF (*r* = 0.793, *p* < 0.001), DDF (*r *= 0.692, *p* < 0.001), and TAS Total (*r* = 0.794, *p* < 0.001). Depression was negatively associated with resilience (*r* = –0.593, *p* < 0.001), whereas no significant association was observed with EOT (*r* = 0.038, *p* = 0.790), age (*r* = 0.213, *p* = 0.133), or BMI (*r* = 0.154, *p *= 0.282).

Resilience (CYRM-12) showed significant negative correlations with DIF (*r* = –0.429, *p* = 0.002), DDF (*r* = –0.581, *p* < 0.001), TAS Total (*r* = –0.536, *p* < 0.001), and depression (*r* = –0.593, *p* < 0.001). A weaker negative correlation was observed with anxiety (*r *= –0.328, *p* = 0.019) (see Table 5).

**Table 5.** **Correlation matrix among study variables in the ED group (*n* = 51)**.

Variable	1	2	3	4	5	6	7	8	9
1. WFIRS-P total	—								
2. DIF	0.326*	—							
3. DDF	0.413**	0.701***	—						
4. EOT	0.135	0.023	0.005	—					
5. TAS total	0.410**	0.921***	0.860***	0.262	—				
6. CYRM-12	−0.308*	−0.429**	−0.581***	−0.095	−0.536***	—			
7. RCADS-CV depression	0.463***	0.793***	0.692***	0.038	0.794***	−0.593***	—		
8. RCADS-CV anxiety	0.391**	0.674***	0.511***	0.036	0.643***	−0.328*	0.708***	—	
9. Age	0.105	0.096	0.083	−0.250	0.033	−0.288*	0.213	0.049	—
10. BMI	0.222	0.008	0.017	−0.057	−0.002	−0.070	0.154	0.030	0.441**

Note. *p < 0.05, **p < 0.01, ***p < 0.001. Reported *p*-values were adjusted for multiple comparisons using the Benjamini–Hochberg False Discovery Rate (FDR) procedure.

#### 3.2.3 Backward Linear Regression Predicting Functional Impairment (WFIRS-P Total) in Adolescents With ED 

A backward multiple linear regression analysis was conducted to examine whether alexithymia subdimensions (DIF and DDF), depressive symptoms (RCADS-CV Depression), anxiety symptoms (RCADS-CV Total Anxiety), and resilience (CYRM-12) explain overall functional impairment as measured by the WFIRS-P Total score in adolescents with EDs (*n* = 51). Multicollinearity was within acceptable limits throughout the models, with VIF values ranging from 1.00 to 4.16. The Durbin-Watson statistic was 1.82, indicating no serious autocorrelation issues. Assumptions of normality (Shapiro-Wilk *p *= 0.145), linearity, and homoscedasticity were met.

The initial model including all five predictors was significant, F_5,45_ = 3.15, *p* = 0.016, accounting for 26% of the variance in functional impairment (*R*^2^ = 0.259, Adjusted *R*^2^ = 0.177). Through backward elimination, only depressive symptoms remained in the final model. The final model was statistically significant, F_1,49_ = 13.38, *p* = 0.001, explaining 21.4% of the variance (*R*^2^ = 0.214, Adjusted *R*^2^ = 0.198).

In the final model, RCADS-CV Depression emerged as a significant predictor of functional impairment (β = 0.463, *p* = 0.001), indicating that higher depressive symptoms were associated with greater functional difficulties. Other variables, including DIF, DDF, anxiety symptoms, and resilience, were excluded during earlier steps due to non-significant contributions (all *p* > 0.10) (see Table 6).

**Table 6.** **Backward linear regression predicting functional impairment (*n* = 51)**.

Step	Variables	B	SE	β	t	*p*	Tolerance	VIF
1	DIF	–0.013	0.012	–0.268	–1.14	0.262	0.296	3.38
	DDF	0.018	0.015	0.250	1.24	0.221	0.404	2.47
	RCADS-CV Depression	0.010	0.007	0.383	1.46	0.150	0.241	4.16
	RCADS-CV Total Anxiety	0.005	0.006	0.174	0.92	0.364	0.455	2.20
	CYRM-12 (Resilience)	0.000	0.006	0.007	0.04	0.968	0.557	1.80
2	DIF	–0.013	0.011	–0.266	–1.16	0.253	0.305	3.28
	DDF	0.018	0.013	0.248	1.32	0.193	0.457	2.19
	RCADS-CV Depression	0.010	0.006	0.379	1.59	0.118	0.285	3.50
	RCADS-CV Total Anxiety	0.005	0.006	0.175	0.94	0.352	0.464	2.16
3	DIF	–0.010	0.011	–0.209	–0.95	0.349	0.327	3.06
	DDF	0.017	0.013	0.238	1.27	0.209	0.459	2.18
	RCADS-CV Depression	0.012	0.006	0.464	2.12	0.039*	0.335	2.99
4	DDF	0.013	0.013	0.177	1.01	0.317	0.521	1.92
	RCADS-CV Depression	0.009	0.005	0.340	1.94	0.058	0.521	1.92
5	RCADS-CV Depression	0.012	0.003	0.463	3.66	0.001***	1.000	1.00

*Note*. *p < 0.05, ***p < 0.001; Dependent variable: WFIRS-P Total. All variables were entered using the backward elimination method.

#### 3.2.4 Mediation Analysis via General Linear Model 

**Fig. 1.**
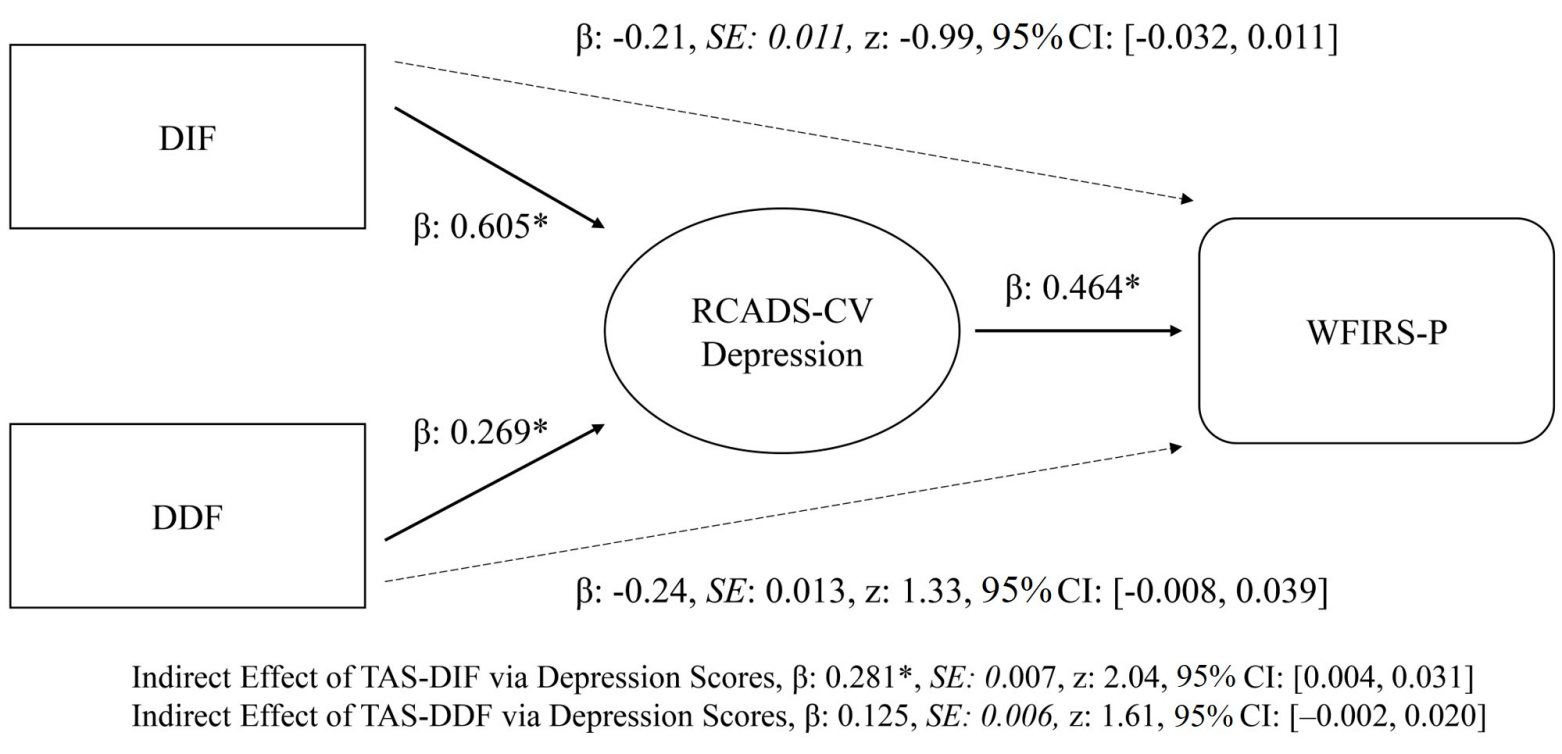
**GLM of mediation analysis**. Confidence intervals were computed with the bias-corrected bootstrap method (5000 resamples). Standardized beta coefficients (β) are fully standardized effect sizes. Dashed lines indicate direct and black lines indicate indirect pathways between variables. β, Standardized Beta value; *SE*, Standard error; *z*, Z-Statistics of GLM model; CI, Confidence interval. *indicates both *p* ≤ 0.05 and confidence interval excludes zero.

A mediation model was tested to explore the indirect association of alexithymia dimensions with functional impairment through depressive symptoms. Using bias-corrected bootstrap estimates (5000 resamples), the indirect effect of DIF on WFIRS-P Total impairment through depressive symptoms was statistically significant (β = 0.281, SE = 0.007, *z* = 2.04, *p* = 0.041, 95% CI [0.004, 0.031]). In contrast, the indirect effect of DDF was not significant (β = 0.125, SE = 0.006, *z* = 1.61, *p *= 0.106, 95% CI [–0.002, 0.020]). Consistent with this pattern, DIF strongly predicted depressive symptoms (β = 0.605, *p* < 0.001), and depressive symptoms were associated with greater impairment (β = 0.464, *p *= 0.027). Although DDF also predicted depressive symptoms (β = 0.269, *p* = 0.018), its pathway to impairment was not significant. Direct effects of DIF and DDF on WFIRS-P Total were nonsignificant (*p*s > 0.05) (see Fig. 1).

## 4. Discussion

The present study compared adolescents diagnosed with an ED to a control group and identified the factors most closely associated with functional impairment. Compared to controls, adolescents with EDs exhibited: (a) significantly higher scores across all alexithymia subscales; (b) marked elevations in internalizing symptoms (depression and anxiety); (c) substantial reductions in psychological resilience; and (d) clinically meaningful impairments in parent-rated functioning. It is important to note that group differences in global functional impairment remained significant after controlling for age, BMI, and sex. Although DDF predicted ED group membership, depressive symptoms emerged as the sole significant contributor to functional impairment within the ED group. Furthermore, DIF was indirectly associated with functional impairment via depressive symptoms, suggesting a potential mediating pathway. A key contribution of this study is its exploration of how alexithymia subdimensions and resilience relate to functional impairment and account for their associations with depression and anxiety. To our knowledge, these findings are novel and offer a meaningful refinement to the existing literature on adolescent EDs.

The primary aim of this study was to map functional impairment in adolescents with EDs and to delineate domain-specific variations in impairment. The greatest between-group disparity was observed in the self-perception domain (Cohen’s *d* = 1.67), followed by life skills (*d* = 0.98), risky behaviors (*d *= 0.85), family relationships (*d *= 0.78), social relationships (*d* = 0.76), and, least affected, school functioning (*d* = 0.52). These domain-level deficits converged in a very large difference on the global composite (*d* = 1.20), which remained robust after adjusting for BMI, age, and sex (η^2^*ₚ *= 0.258). Sensitivity analyses, conducted by excluding participants with clinically significant levels of anxiety or depression, revealed that the main findings not only persisted but also yielded larger effect sizes. This suggested that the observed impairments became even more pronounced when comparisons were limited to a more strictly defined “healthy” control group. Nonetheless, due to the reduced sample size and reliance on exclusion criteria based solely on internalizing symptoms from a self-report scale, these results should be interpreted with caution and ideally replicated in larger samples that are confirmed to be free of psychopathology by standardized diagnostic interviews. Methodologically, no previous study appears to be directly comparable to the present study; however, the findings of the present study are broadly consistent with the heterogeneous literature, despite variations in measurement tools, domains assessed, age ranges, diagnostic composition, and control-group selection.

Not all previous studies specifically focused on functional impairment. But many consistently linked EDs to disrupted self-concept, low self-esteem, and diminished global self-worth, which are core features in theoretical models that conceptualize EDs as self-oriented disorders characterized by intense self-criticism, fragmented identity, and low self-compassion [51, 52, 53, 54]. Notably, the largest between-group difference in the present study was observed in the Self-Perception domain of the WFIRS-P. The mean score for the ED group (*M* = 1.88) not only significantly exceeded that of the control group (*M* = 0.56), but also surpassed the established clinical threshold of 1.5, indicating clinically meaningful impairment. This finding, supported by a very large effect size, suggests that self-perception difficulties are a particularly impaired domain of functioning in adolescents with EDs. These results emphasize the importance of incorporating self-related constructs into both conceptual models and treatment approaches. Interventions targeting self-criticism, deficits in self-compassion, and fragmented self-concept may therefore hold promise for reducing symptom severity and promoting sustained functional recovery [55].

Although comparative studies are limited, studies have indicated greater impairment across family, peer, romantic, and school domains among youth with EDs than among matched peers, which is a pattern that was replicated in the present study [2]. EDs have consistently been associated with disrupted social and family functioning [2, 16, 56, 57, 58]. Population-based surveys further linked ED features to reduced quality of life and heightened psychological distress, and clinical samples have demonstrated that severe ED symptoms result in substantial functional loss [4, 9, 10, 11]. However, it is important to acknowledge that psychosocial impairment and social withdrawal may function as transdiagnostic risk factors for both the onset and maintenance of EDs, which suggests potential bidirectional relationships and leads to caution against causal interpretations [5, 59]. When evaluating functional impairment in risky behaviors, a large effect-size difference was again observed between groups. Previous research has linked EDs to a range of risky behaviors, including risky sexual activity, substance use, suicide and non-suicidal self-injury, and impulsivity, particularly in individuals with BED [60, 61, 62, 63, 64, 65]. Therefore, beyond the internalizing features of EDs, their association with risky behavioral tendencies warrants careful consideration.

Although some previous reports suggested that school performance in adolescents with EDs may remain relatively intact [66, 67], the findings of the present study indicated a different pattern. In our sample, school functioning was significantly lower in the ED group than in controls. Notably, 17.6% of participants in the ED group, all diagnosed with anorexia nervosa, required hospitalization in a pediatric ward for medical stabilization and re-nutrition prior to psychiatric consultation. The inclusion of these more severe inpatient cases, treated in a tertiary care setting, likely contributed to the greater impairments observed in school functioning. However, due to the limited sample size, the available data did not permit analysis of functional outcomes by specific diagnostic subgroups or inpatient status. Furthermore, given the strong associations between alexithymia, functional impairment, and ED severity, the absence of a direct measure of illness severity in the present study necessitated a cautious interpretation of the results [22, 23, 24, 25].

The second aim of the present study was to investigate the factors associated with functional impairment within the ED group, alongside affective symptoms. In this cohort, functional impairment was significantly correlated with five variables: DIF (*r *= 0.326, *p* = 0.020); DDF (*r *= 0.413, *p *= 0.003); lower resilience (r = –0.308, *p* = 0.028); depressive symptoms (*r* = 0.463, *p* < 0.001); and anxiety symptoms (*r* = 0.391, *p* = 0.005). However, backward stepwise regression analysis revealed that depression was the sole independent predictor of functional impairment, underscoring its central role in adolescent ED-related disability. Although most available data were derived from adult samples, previous research has consistently demonstrated that depression is a key factor in explaining functional impairment in EDs across both clinical and community populations [13, 68]. Although no study has directly examined this relationship in adolescents, evidence has suggested that EDs tend to present more severely when comorbid with depression, particularly when accompanied by anxiety [29]. Moreover, a prospective study in a high-risk sample of female military college students, an age group comparable to the current cohort, found that functional impairment predicted subsequent depressive symptoms, further emphasizing the close link between depression and functional loss [15]. Taken together, these findings indicate the importance of prioritizing depressive symptoms as an early developmental target, rather than focusing exclusively on the behavioral manifestations of EDs. However, the limited sample size in the present study precluded analyses by diagnostic subgroup or inpatient status. Given the established associations between alexithymia, functional impairment, and illness severity, the absence of a direct assessment of illness severity warrants cautious interpretation of these findings.

A key finding of this study is that DIF was associated with functional impairment indirectly through depressive symptoms, whereas DDF was not. This result partially reflects meta-analytic evidence showing moderate associations between DIF/DDF and depression, but only weak links with EOT. This result closely resembles the demonstration by Rice *et al*. (2022) [23] that DIF occupies a central role in the alexithymia → depression → disordered-eating-attitudes pathway in populations at risk for BED and obesity [28]. Similarly, analyses based on total alexithymia scores have shown that alexithymia directly contributes to elevated ED symptoms and is also indirectly associated with ED symptoms via increased depressive symptoms [69]. These findings suggest that DIF may function as a transdiagnostic “active ingredient”, whereby higher DIF intensifies depressive symptoms, which, in turn, undermine functional capacity. This cascade of effects aligned with adult studies that linked elevated DIF/DDF levels to suicidal ideation and their strong associations with low self-directedness and poor interoceptive awareness [70, 71]. Developmentally, heightened DIF has been linked to early interpersonal trauma and chronic emotional neglect, and may even predict treatment outcomes independently of depression or ED severity [22, 72]. Therefore, DIF appears to represent a pivotal factor in the interplay between EDs and depressive symptoms. These findings offer a more nuanced understanding of how DIF and DDF differentially relate to depression in EDs and underscore the need for further research into shared etiological mechanisms, particularly within the domains of trauma and neurobiology, to inform more effective prevention and treatment strategies.

Consistent with previous research, the ED group scored significantly higher than controls on total alexithymia and its subscales [19, 20, 25]. The finding that the mean alexithymia score in the ED group exceeded the scale’s established cut-off was particularly noteworthy. This indicates the importance of considering alexithymia as a critical domain for screening and intervention, not only based on group comparisons, but also due to scores surpassing clinically significant thresholds. Both core facets of alexithymia, DIF and DDF, are reliably associated with ED symptoms; meta-analytic evidence confirmed markedly elevated DIF, DDF, and EOT in individuals with EDs [19]. In the data of the present study, however, only DDF remained a unique discriminator of group status once covariates were controlled, suggesting that expressive aspects of alexithymia, rather than difficulties in emotion recognition, may be more closely linked to core ED pathology in adolescents, even after accounting for anxiety and depressive symptoms. This observation agreed with and extended previous findings in adolescent populations, highlighting DIF and DDF as the alexithymia dimensions most specific to EDs and, therefore, prime targets for emotion-focused interventions. Evidence from treatment studies may be promising in this regard [20, 25, 73]. Internalizing symptoms also followed the expected pattern: anxiety and depression were markedly elevated in adolescents with EDs in our sample, which was consistent with recent epidemiological estimates [30, 74]. Given their strong correlation with functional impairment, particularly in relation to depressive symptomatology, these affective disturbances warrant close screening alongside weight and behavioral indicators.

Adolescents diagnosed with EDs demonstrated significantly lower levels of psychological resilience than did controls, aligning with robust evidence that showed that reduced resilience constitutes a notable vulnerability within the ED population [36, 37, 40, 75, 76]. Although correlation analyses revealed a significant inverse relationship between functional impairment and resilience (*r *= –0.308, *p* = 0.028), resilience did not emerge as an independent predictor of functional impairment in the regression model. This suggested that its influence on functional outcomes may have been limited in this specific context. The absence of a significant effect may have indicated that the protective role of resilience unfolds gradually over time, is mediated by unmeasured factors (e.g., social support, family cohesion, treatment engagement), or that conventional trait-based assessments fail to adequately capture the subjective and context-sensitive aspects of resilience that buffer against functional impairment. Notably, to the best of our knowledge, the present study is the first in the literature on adolescent ED to examine both group-level differences and the association between resilience and functional impairment. Therefore, these findings should be interpreted with caution, and future longitudinal research is needed to replicate the results and elucidate the dynamic and potentially indirect role of resilience in everyday functioning.

This study presented several important clinical implications. The pronounced functional impairment that was observed highlighted the necessity of routine, multi-informant functional screening in adolescents with EDs. Early identification of deficits in self-perception and depressive symptoms may be crucial for preventing the onset of future mood disorders, illness chronicity, and suicidality [15, 23]. Given that residential treatment only partially alleviates alexithymia, systematic assessment of its two core dimensions, particularly DIF, is strongly recommended due to its strong association with depression risk [25]. Targeted emotion-focused interventions, such as cognitive remediation and emotional skills training (CREST) or emotion-focused cognitive behavioral therapy/emotion acceptance behavior therapy, should be implemented at early stages. Complementary models, including Maudsley model anorexia nervosa treatment for adolescents and young adults (MANTRa) and radically open dialectical behavior therapy, may further enhance long-term outcomes [77, 78, 79, 80]. Rehabilitation strategies should also address domain-specific deficits: self-compassion and identity-based therapies may improve self-perception; school-based counseling can help mitigate academic difficulties; and structured monitoring is essential for managing risky behaviors. Finally, incorporating resilience-building modules into ED treatment protocols may provide enduring protective effects.

This study demonstrated several noteworthy methodological strengths that enhance its ecological validity and its contribution to the existing literature. The inclusion of clinically diagnosed cases of EDs, rather than subclinical or community-based samples, strengthens both the validity and clinical relevance of the findings. The use of clinician-confirmed diagnoses, parent-reported assessments of functional impairment, and a comprehensive functional inventory minimized single-informant bias and offered a multidimensional perspective on functioning. Moreover, rigorous statistical control for multiple covariates further reinforced the robustness of the results. Sensitivity power analyses confirmed that the available sample size was sufficient to detect medium-to-large effects in both group comparisons and ANCOVA models. Notably, most observed effects exceeded these thresholds, supporting the adequacy of the sample for testing the primary hypotheses.

Nevertheless, this study had several limitations. The modest sample size and uneven distribution of diagnostic subgroups may have affected the stability of parameter estimates and limited the generalizability of the findings. Additionally, the cross-sectional design precluded any conclusions regarding causality, and the aggregation of different ED subtypes into a single group, although consistent with previous research on the transdiagnostic nature of alexithymia, represented a clear limitation, as it may have obscured clinically meaningful differences across specific diagnostic categories. Sex differences were not examined within a comparative framework; however, previous studies suggested that functional impairment tends to be largely comparable across genders [11]. Moreover, the absence of formal symptom-severity measures constituted a potential confound, and reliance solely on parent-reported functioning may have resulted in under- or overestimation of impairment, particularly in contexts involving parent–adolescent conflict or parental psychopathology [58]. Furthermore, illness-course characteristics (e.g., acute, remitted, or chronic presentations) were not systematically assessed. Another notable limitation concerned the control group, which was recruited from siblings of patients attending the same outpatient clinics (excluding those with EDs). Although this approach ensured comparable sociodemographic backgrounds between groups, it may have introduced biases that warrant cautious interpretation. Additionally, the lack of standardized psychiatric interviews raised the possibility of undetected subthreshold symptoms. Given these limitations, the findings should be interpreted with caution, and the novel results require replication in future studies.

Future research would benefit from prospective, longitudinal cohort designs with larger, diagnostically stratified samples to elucidate the temporal relationships among alexithymia, depressive symptoms, and functional impairment. Incorporating standardized measures of illness severity, together with stratification by diagnostic subgroups and inpatient versus outpatient status, could facilitate a more refined understanding of how clinical heterogeneity and level of care shape these associations. Broader, multi-site, or community-based recruitment strategies may also enhance the generalizability of findings. Additionally, integrating multi-method approaches—including physiological indicators, such as the cortisol awakening response or heart-rate variability—may help clarify underlying mechanisms. Finally, intervention studies examining whether improvements in emotion recognition, emotional expression, or resilience translate into sustained functional gains would provide valuable insights for clinical practice.

## 5. Conclusion

The present study indicated that adolescents with EDs exhibit pervasive functional impairments that are closely associated with depressive symptomatology. Whereas the difficulty in describing feelings was more strongly related to ED diagnosis, the difficulty in identifying feelings showed an indirect association with functional impairment via depressive symptoms. Early, targeted interventions that enhance emotional awareness and expressive abilities may therefore be pivotal in improving not only depressive symptoms but also everyday functional outcomes. Equally important, the pronounced deficits in self-perception underscore this domain as a key therapeutic target. Taken together, the findings supported the routine integration of multi-informant functional assessments alongside systematic screening for alexithymia and depressive symptoms in adolescents with EDs. Future longitudinal, multimodal research, including neurobiological indices, should be used in order to clarify the dynamic contributions of emotion-identification deficits, depressive affect, and resilience to functional outcomes across ED subtypes.

## Availability of Data and Materials

The data that support the findings of this study are available on reasonable request from the corresponding author. The data are not publicly available due to restrictions containing information that could compromise the privacy of research participants.

## Supplementary Material

Supplementary material associated with this article can be found, in the online version, at https://doi.org/10.31083/AP45458.
