# Supplementary material for: Alexithymia, Resilience, and Depression in Functional Impairment Among Adolescents With Eating Disorders: A Case-Control Study
Source: Alpha Psychiatry. 2026 Feb 26;27(1):45458. doi: 10.31083/AP45458 (PMC12957964; doi:10.31083/AP45458)
Supplement: Supplementary file 1 [file 2757-8038-27-1-45458-s1.zip › Supplementary Material.docx]

**Supplementary Material**

### **S1. Detailed Psychometric Properties**

**Toronto Alexithymia Scale (TAS-20)**

- **Factor structure:** Confirmatory factor analyses supported the original three-factor model (DIF, DDF, EOT). 31% of total variance explained with 3-factor solution
- **Internal consistency:** Cronbach’s α = .78 (Total). .78 (DIF), .75 (DDF), .66 (EOT)
- **Test–retest reliability:** r = .81.
- **Convergent validity:** Significant correlations with depression and anxiety scales.
- **Turkish adaptation:** Adaptation study held with 390 undergraduate or post-college graduate students. Original three-factor solution were confirmed. 34.9% of total variance explained with 3-factor solution. Total Cronbach’s alpha value was found .78. DIF, DDF and EOT Factor Cronbach’s alpha levels were .80,.57 and .63, respectively.

**Child and Youth Resilience Measure – 12 (CYRM-12)**

- **Factor structure:** Original version of CYRM have 28 questions and 4 factors . Shortened 12-question version have confirmed single-factor solution. Factor loadings varied between .39-.89. Confirmatory factor analysis have shown satisfactory fit with (χ2 (51, N=1540) = 255.419, p=0.0001; Adjusted Goodness of Fit Index = 0.960; Comparative Fit Index = 0.957; Root Mean Square Error of Approximation = 0.050).
- **Internal consistency:** Cronbach’s α = .84.
- **Validity:** Significant correlations with self-efficacy, positive affect, and negative affect.
- **Turkish adaptation:** Adaptation study held with 256 mid- and high-school students aged between 11-16. Single-factor solution were confirmed with %51.3 of the total variance explained. Factor loadings varied between .54-.81. Cronbach’s alpha value was found .91

**Revised Child Anxiety and Depression Scale – Child Version (RCADS-CV)**

- **Factor structure:** 6-factor solution have found in Exploratory factor analysis. Factors labeled as Social Phobia, Panic Disorder and Agoraphobia, Major Depressive Disorder, Seperation Anxiety Disorder, Generalized Anxiety Disorder and Obsessive-Compulsive Disorder. 6-factor solution explained 41.3% of total variance. Factor loadings vary between .27-.64
- **Internal consistency:** Cronbach’s α = .95 (total); α = .73–.82 (subscales).
- **Validity:** Convergent validity supported by correlations with other depression and anxiety scales..
- **Turkish adaptation:** Adaptation study held with 483 children aged 8-17. Cronbach’s alpha value have found .95 for total scale, .75-.86 for subscales. Confirmatory factor analysis supported the original six-factor model with RMSEA: .053, goodness-of-fit index .82, CFI: .85, TLI: .84. Factor loadings varied .31 to .80. ROC Analyses shown .67 sensitivity and .72 specifity values.

**Weiss Functional Impairment Rating Scale – Parent Report Form (WFIRS-P)**

- **Factor structure:** Six factor-solution (Family, School, Life Skills, Self-Concept, Social, Risky Activities). 4-week test-rerest correlation values were found >.7.
- **Internal consistency:** Cronbach’s α = .93 (total); subscales range .56–.89.
- **Validity:** Associations with emotional/behavioral measures support validity.
- **Turkish adaptation:** Adaptation study held with parents of 500 children (250 diagnosed with ADHD and 250 control group). Exploratory factor analysis shown 7-factor solution with 54.5% of total variance explained. Confirmatory factor analyses shown satisfactory fit with CFI: .95, RMSEA: .061. Cronbach’s alpha value of total scale were found .93. Cronbach’s alpha values of subscales were .56-.93. 4-week interval test-retest correlation values of subscales were found .79-.93.

**S2. Sensitivity Analyses**

To address potential misclassification in controls, we repeated all analyses after excluding control participants with RCADS-CV total T-scores ≥70. False discovery rate (FDR; Benjamini–Hochberg) corrections were applied to account for multiple comparisons.

**Table 1. Independent samples t-tests (RCADS ≥ 70 excluded)**

| **Variables** | **t(df)** | **Mean diff** | **95% CI** | **p (raw)** | **q (FDR)** | **d** |
| --- | --- | --- | --- | --- | --- | --- |
| **WFIRS-P Family** | 4.59(88) | 0.43 | 0.25 – 0.62 | <.001** | <.001** | 0.98 |
| **WFIRS-P School** | 3.13(88) | 0.29 | 0.10 – 0.47 | .002** | .003** | 0.67 |
| **WFIRS-P Life Skills** | 5.25(88) | 0.54 | 0.33 – 0.74 | <.001** | <.001** | 1.12 |
| **WFIRS-P Self-Perception** | 9.27(88) | 1.52 | 1.19 – 1.84 | <.001** | <.001** | 1.97 |
| **WFIRS-P Social Activities** | 4.35(88) | 0.49 | 0.26 – 0.71 | <.001** | <.001** | 0.93 |
| **WFIRS-P Risky Behaviors** | 4.56(88) | 0.16 | 0.09 – 0.22 | <.001** | <.001** | 0.97 |
| **WFIRS-P Total** | 6.93(88) | 0.44 | 0.31 – 0.57 | <.001** | <.001** | 1.47 |
| **TAS–DIF** | 7.92(88) | 10.80 | 8.09 – 13.52 | <.001** | <.001** | 1.68 |
| **TAS–DDF** | 7.11(88) | 6.92 | 4.98 – 8.85 | <.001** | <.001** | 1.51 |
| **TAS–EOT** | 2.46(88) | 1.83 | 0.35 – 3.30 | .016* | .021* | 0.52 |
| **TAS Total** | 8.28(88) | 19.55 | 14.86 – 24.24 | <.001** | <.001** | 1.76 |
| **CYRM-12** | –5.97(88) | –11.58 | –15.43 – –7.73 | <.001** | <.001** | –1.27 |
| **RCADS Depression** | 7.78(87) | 22.75 | 16.94 – 28.56 | <.001** | <.001** | 1.67 |
| **RCADS Anxiety** | 7.22(86) | 17.74 | 12.86 – 22.63 | <.001** | <.001** | 1.56 |
| **RCADS Total T** | 7.91(86) | 19.90 | 14.89 – 24.90 | <.001** | <.001** | 1.71 |

*Note.* ED = 1, Control = 0. d = Cohen’s d. *p < .05, **p < .01 after FDR correction.

| **Table 2. ANCOVA Analysis of WFIRS-P Total Scores** | | | | | | | |
| --- | --- | --- | --- | --- | --- | --- | --- |
|  | **Sum of Squares** | **df** | **Mean Square** | **F** | **p** | **η²** | **η²p** |
| **Overall model** | 4.64437 | 4 | 1.16109 | 12.8592 | <.001 |  |  |
| **Group** | 4.07534 | 1 | 4.07534 | 45.9594 | <.001 | 0.345 | 0.362 |
| **Age** | 0.00332 | 1 | 0.00332 | 0.0375 | 0.847 | 0.000 | 0.000 |
| **BMI** | 0.21844 | 1 | 0.21844 | 2.4634 | 0.120 | 0.018 | 0.030 |
| **Sex** | 0.34727 | 1 | 0.34727 | 3.9164 | 0.051 | 0.029 | 0.046 |
| **Residuals** | 7.18247 | 81 | 0.08867 |  |  |  |  |
